# Synergistic effect of selected carboxylic acids and phenolic compounds detected by the FRAP method

**DOI:** 10.1016/j.fochx.2024.101573

**Published:** 2024-06-17

**Authors:** Petra Švestková, Josef Balík, Ivo Soural

**Affiliations:** Department of Post-Harvest Technology of Horticultural Products, Faculty of Horticulture, Mendel University in Brno, Lednice, Czech Republic

**Keywords:** FRAP (ferric reducing antioxidant power), Synergistic effect, Antioxidants, Polyphenols, Carboxylic acids, Methoxyphenols

## Abstract

Antioxidants in nutrition are a widely discussed topic. In this study, a synergistic effect was observed for 13 selected substances – antioxidants and potential synergists, whereby two substances were mixed in the same concentration ratio of 1:1. The antioxidant capacity (AC) of the mixtures was determined using the FRAP method. The AC measured was compared with a theoretical AC value (as only additive effect) to calculate the synergistic or antagonistic effect. Out of 78 possible combinations, a synergistic effect (SE) was detected in 72. For the 10 combinations, the SE was more than twice that of the pure substances. The largest synergistic effect was exhibited by vanillin and 4-hydroxybenzoic acid with increases even above 200% compared to the pure substances. Some of the phenolic substances that were subject to measurement can be used for the fortification of fruit juices.

## Highlights

• Synergistic effect on AC up to 8.9-fold for food compounds.
• Increase in AC of real mixtures by up to 5.5 mM Trolox (ie +85% vs. hypothetical).
• Compounds with low AC showed higher synergistic effects and vice versa.
• Synergists improve the AC of real food.

## 1 Introduction

An antioxidant is a substance that slows down, stops or directly removes oxidative damage to a molecule, e.g. by binding excess free radicals to itself (Sonam & Guleria, 2017). Antioxidants in the body act as anti-inflammatory agents and help slow down the aging process and reduce the risk of serious diseases such as cancer, cardiovascular diseases, etc. Antioxidants are abundant in fruits (apples, raspberries, blackberries, currants and others), vegetables (onions, cabbage, celery, parsley and others), legumes, spices, herbs, teas and oils (Dolas Ashadevi & Gotmare, 2015; Motooka et al., 2015). The amount of antioxidants and antioxidant activity depends on the climatic and soil conditions, growing and harvesting conditions and plant variety and morphological part. Free radicals are reactive types of substances with unpaired electrons. They occur as reactive oxygen species (ROS), nitrogen species (RNS) and chlorine species (Flieger et al., 2021). The most common are ROS, which are the result of cellular metabolism and can damage some cellular structures (lipids, proteins, saccharides, etc.) and can alter their function. If the presence of ROS is higher than of antioxidants, a condition referred to as oxidative stress occurs (Birben et al., 2012). Radicals and antioxidants are considered as an antagonistic pair, both in balance are necessary for the proper functioning of the organism (Nikolaidis & Margaritelis, 2023). Individual antioxidants act through multiple mechanisms based on the reaction system and, in addition, respond differently to different sources. Therefore, there is no single universal method that would present a comprehensive picture of antioxidants (Prior et al., 2005). FRAP (Ferric Reducing Antioxidant Power) is one of the evaluation methods, which is based on the reduction of ferric ion to a divalent ion; this method has been known for over 20 years (Benzie & Strain, 1996) and can be encountered in the literature in various slight modifications (Guo et al., 2003; Thaipong et al., 2006). The standard reaction time is 10 min, but this time is not enough for some antioxidants (Munteanu & Apetrei, 2021). Antioxidant capacity (AC) is the summative property of many substances found together in a given food; antioxidants commonly include phenolic/polyphenolic substances, some of the vitamins, etc. (Martinović et al., 2010). The substances can interact additively, antagonistically or synergistically (Wang et al., 2011), in other words only the sum of AC, negative or positive synergistic effect. Substances referred to as antioxidant synergists are substances with minimal or no antioxidant capacity, but capable of increasing the antioxidant capacity of some antioxidants. A synergistic effect occurs when the resulting value is greater than the sum of each of the acting elements individually – additive effect (Sonam & Guleria, 2017). An antagonistic effect is when the effect of two combined components is less than the sum of the effects of each component used individually (Olszowy-Tomczyk, 2020). Carboxylic acids – e.g. citric, tartaric and others – are also considered among such substances (Vicol, 2022). The aim of this work was to evaluate the mutual synergistic effect of 13 selected substances, members of antioxidants and potential synergists – that are frequently found in foods. It is hypothesized that the synergist, under appropriate conditions, can extremely enhance the antioxidant capacity.

## 2 Materials and method

### 2.1 Chemicals

A total of 13 substances were monitored in the determination of the synergistic effect, of which four were carboxylic acids: ascorbic acid (99% Sigma-Aldrich, Germany), citric acid (≥ 99% Sigma-Aldrich, Germany), malic acid (≥ 97% Sigma-Aldrich, Japan), tartaric acid (99.5% Sigma-Aldrich, Spain); 3 methoxyphenols: eugenol (≥ 99% Sigma-Aldrich, Indonesia), vanillin (≥99%, Alfa Aesar, Germany), guaiacol (≥ 99% Acros, China); and six phenolic acids; for the latter, three were benzoic acid derivatives: 4-hydroxybenzoic acid (≥ 99% Sigma-Aldrich, China), protocatechuic acid (≥ 97% Alfa Aesar, Germany), gentisic acid (≥ 99% Alfa Aesar, Germany); and three were cinnamic acid derivatives: chlorogenic acid (≥ 99% Alfa Aesar, Germany), caffeic acid (98%, Sigma-Aldrich, China), syringic acid (95%, Sigma-Aldrich, Great Britain).

Other substances used in the analysis were as follows: Acetic acid (99.99%, Sigma-Aldrich, USA), Ethanol absolute (>99.7%, VWR Chemicals, France), Hydrochloric acid 35% (p.a., Ing. Petr Švec - PENTA, Czech Republic), Methanol (99.9%, Sigma-Aldrich, Germany), Iron(III) chloride (97%, Sigma-Aldrich, Germany), Sodium acetate trihydrate (p.a., Ing. Petr Švec - PENTA, Czech Republic), TPTZ (2,4,6-tris(2-pyridyl)-*s*-triazine) (99%, Acros Organics, China), Trolox (6-hydroxy-2,5,7,8-tetramethylchroman-2-carboxylic acid) (98%, Sigma-Aldrich, Germany).

### 2.2 Method FRAP

The reaction solution for FRAP method was composed of three parts (1:1:10 ratio). The solution of FeCl_3_ was the first one; prepared by weighing out and dissolving 0.081 g of FeCl_3_.6H_2_O in a 25 ml volumetric flask adding de-ionised water up to the punch mark. The second part was a 25 ml solution consisting of 0.078 g of TPTZ, 88.25 μl of 35% HCl and de-ionised water. The third part of the reaction solution was acetate buffer (pH: 3.6), which was obtained by pipetting 4 ml of concentrated CH_3_COOH and weighing 0.775 g of CH_3_COONa.

Trolox solution of five different concentrations (0.1; 0.2; 0.3; 0.4 and 0.5 mmol.l^−1^) was used for calibration.

The analysed samples were prepared in a 50 ml volumetric flask with 0.1000 g of antioxidant (precision of 0.0001 g), 10 ml of ethanol (pipetted) and the third part de-ionised water added up to the punch mark. All substances had a final concentration of 2 g/l.

For the measurement, 2000 μl of FRAP reaction solution and 25 μl of diluted sample were pipetted into a 1 cm thick cuvette. Subsequently, the cuvette was stirred for 10 s with a shaker and placed in the dark for a reaction time of 10 min. After the reaction time, the cuvettes were placed in a spectrometer (Specord 50 PLUS) and analysed at a wavelength of 593 nm. The method has also been used in previous studies (Híc et al., 2017; Soural et al., 2020). All measurements were performed at a laboratory temperature of 21 °C. All antioxidant capacities of diluted samples were recalculated to a final concentration 2 g/l.

### 2.3 Measuring the synergistic effect

To determine the synergistic effect, the two substances were mixed in a concentration ratio of 1:1, i.e. 1 g/l concentration of one substance and 1 g/l of the other substance, where the total final concentration was 2 g/l each time. The solution was determined by the FRAP method in the same way as pure solutions of individual substances. The measured antioxidant capacity (AC) of the mixture of the two substances was compared with the theoretical value (sum of half of the first substance with half of the second substance); if the measured value of the mixture showed a higher AC, it involved a synergistic effect.

The absolute magnitude of the synergistic effect (mM) was converted to multiples of the increase corresponding to the percentage increase in AC (Table 1); it was done against a theoretical additive effect each time so that individual substances with different AC could be compared with each other and the synergistic effect could be expressed regardless of the size of the concentration. At the same time, the statistical percentage increase in AC was calculated.

**Table 1:** Antioxidant capacity (AC) of pure substances of 2 g/l concentration – of each of their binary mixtures – in mM Trolox equivalent. Synergistic effect as percentage increase in AC (%) for real mixtures relative to the theoretical mixture without this effect (where statistically significant changes are indicated by “*” and statistically non-significant changes by “n. s.”).

		{1}	{2}	{3}	{4}	{5}	{6}	{7}	{8}	{9}	{10}	{11}	{12}	{13}
Compound		Ascorbic a.	Malic a.	Citric a.	Tartaric a.	4-hydroxybenzoic a.	Caffeic a.	Eugenol	Vanillin	Guaiacol	Protocatechuic a.	Chlorogenic a.	Gentisic a.	Syringic a.
AC (mM)		12.83 ± 0.16	0.31 ± 0.08	0.29 ± 0.07	0.32 ± 0.06	0.19 ± 0.13	12.36 ± 0.44	23.60 ± 0.26	0.58 ± 0.07	25.87 ± 0.08	15.71 ± 0.24	7.01 ± 0.09	27.44 ± 0.17	16.87 ± 0.13
{1}	mM		6.53 ± 0.24	6.27 ± 0.48	7.38 ± 1.03	6.35 ± 0.57	13.25 ± 0.34	19.00 ± 0.74	6.31 ± 0.50	19.93 ± 0.42	14.67 ± 0.17	9.67 ± 0.55	19.51 ± 0.27	15.17 ± 0.18
	%		−0.7%	−4.5%	+12.3%	−2.4%	+5.2%	+4.3%	−5.9%	+3.0%	+2.8%	−2.5%	−3.1%	+2.2%
{2}	mM	n. s.		0.76 ± 0.13	0.82 ± 0.13	0.72 ± 0.12	7.39 ± 0.31	12.46 ± 0.18	0.90 ± 0.06	13.69 ± 0.17	8.62 ± 0.12	4.21 ± 0.13	14.45 ± 0.05	9.23 ± 0.07
	%			+154.5%	+162.8%	+189.2%	+16.7%	+4.2%	+104.0%	+4.6%	+7.7%	+14.9%	+4.2%	+7.5%
{3}	mM	n. s.	*		1.11 ± 0.05	1.21 ± 0.11	7.49 ± 1.13	12.98 ± 0.24	1.39 ± 0.22	13.95 ± 0.06	9.04 ± 0.10	4.69 ± 0.08	14.93 ± 0.12	9.65 ± 0.13
	%				+263.5%	+400.8%	+18.4%	+8.7%	+218.7%	+6.6%	+12.9%	+28.5%	+7.7%	+12.5%
{4}	mM	*	*	*		2.28 ± 1.60	8.64 ± 0.46	13.23 ± 0.19	1.60 ± 0.23	14.43 ± 0.50	9.19 ± 0.24	4.94 ± 0.16	15.54 ± 0.88	9.74 ± 0.27
	%					+789.9%	+36.3%	+10.6%	+256.2%	+10.2%	+14.7%	+34.7%	+12.0%	+13.3%
{5}	mM	n. s.	*	*	*		9.30 ± 0.18	14.70 ± 0.81	2.05 ± 0.21	15.95 ± 1.53	9.51 ± 0.10	5.23 ± 0.21	16.71 ± 0.94	10.85 ± 1.01
	%						+48.1%	+23.6%	+429.4%	+22.4%	+19.6%	+45.3%	+21.0%	+27.2%
{6}	mM	*	*	n. s.	*	*		21.56 ± 0.19	11.97 ± 2.60	23.36 ± 1.14	17.68 ± 0.46	13.48 ± 0.19	24.06 ± 0.85	18.58 ± 0.44
	%							+19.9%	+85.0%	+22.2%	+26.0%	+39.1%	+20.9%	+27.1%
{7}	mM	n. s.	*	*	*	*	*		14.62 ± 0.27	27.90 ± 0.18	21.90 ± 2.41	17.71 ± 0.73	29.07 ± 1.31	22.71 ± 0.40
	%								+20.9%	+12.8%	+11.4%	+15.7%	+13.9%	+12.2%
{8}	mM	n. s.	*	*	*	*	*	*		15.39 ± 0.24	10.10 ± 0.10	5.64 ± 0.05	16.05 ± 0.07	10.66 ± 0.10
	%									+16.3%	+23.9%	+48.4%	+14.6%	+22.1%
{9}	mM	*	*	*	*	*	*	*	*		23.24 ± 0.75	18.75 ± 0.20	28.47 ± 1.45	24.18 ± 1.93
	%										+11.8%	+14.1%	+6.8%	+13.2%
{10}	mM	*	*	*	*	*	*	n. s.	*	*		13.19 ± 0.80	23.33 ± 0.75	19.33 ± 0.85
	%											+16.1%	+8.1%	+18.6%
{11}	mM	n. s.	*.	*	*	*	*	*	*	*	*		19.34 ± 1.41	14.07 ± 0.87
	%												+12.3%	+17.8%
{12}	mM	*	*	*	*	*	*	*	*	*	*	*		23.48 ± 1.56
	%													+6.0%
{13}	mM	n. s.	*	*	*	*	*	*	*	*	*	*	n. s.	
	%													

### 2.4 Statistics

**Fig. 1:**
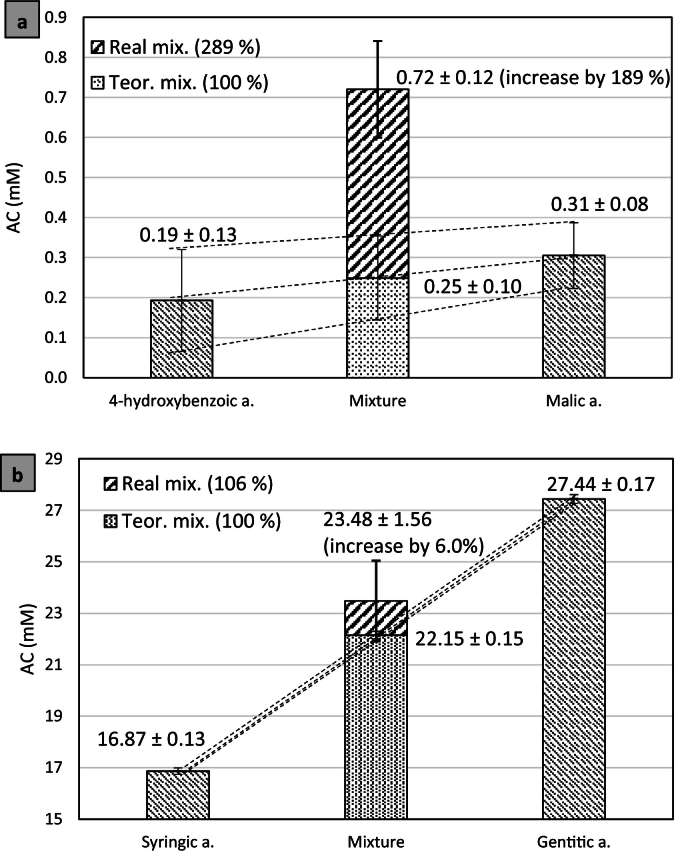
Representation of the calculation of the synergistic effect in mM and determination of statistical significance using the example of combinations of compounds, (a) 4-hydroxybenzoic acid and malic acid (as statistically significant, “*”), (b) gentistic acid and syringic acid (as statistically non-significant, “n. s.”); at concentration 2 g/l.

Due to the impossibility of practically measuring the theoretical AC value of the two-component mixture without synergistic effect, the ratio of both pure substances was used; the mean and standard deviation of three measurements were calculated for one substance, and the same was applied to the other substance. The sum of ½ of one substance and ½ of the other substance was used as the theoretical value (as only additive effect), where the standard deviation of the theoretical value was also the sum of ½ standard deviation of the first substance and ½ standard deviation of the second substance. Such calculated value with calculated standard deviation was compared with the actually measured (real) value of the mixture and its standard deviation from three measurements (Fig. 1).

**Fig. 2:**
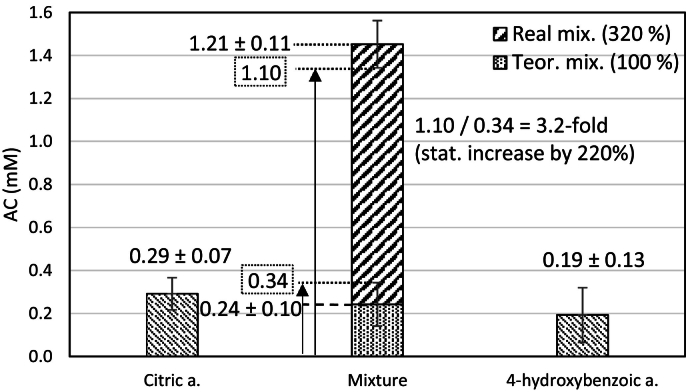
Representation of the calculation of the statistical increase of 220%, at concentration 2 g/l.

In addition to the statistically significant increase in AC (Fig. 1A vs. 1B) a statistical percentage increase was observed, i.e. an increase greater than the measurement error (Fig. 2) when even after subtracting the standard deviation from the average value of the real mixture, the value of the real mixture was 10%, 25% or 100% greater than the average theoretical value (as only additive effect) with standard deviation. For example, the combination of citric acid with 4-hydroxybenzoic acid: 1.21 – 0.11 = 1.10 mM for real mixture; VS. 0.24 + 0.10 = 0.34 mM for the theoretical mixture; their ratio of 1.10 mM / 0.34 mM = 3.2-fold, this corresponds to a statistical increase of 220% (Fig. 2).

## 3 Results and discussion

### 3.1 Antioxidant capacity of pure substances

In Trolox equivalents, the carboxylic acids had the lowest antioxidant capacity (AC): citric acid (0.29 mM), malic acid (0.31 mM) and tartaric acid (0.32 mM), except ascorbic acid (12.83 mM); the lowest AC in general was found for 4-hydroxybenzoic acid (0.19 mM). A low value was also shown for vanillin (0.58 mM), which still did not reach 8 mM (equivalent of 2 g/l Trolox with an identical concentration).

On the other hand, for the range of cinnamic acids, chlorogenic acid (7.01 mM), caffeic acid (12.36 mM) and syringic acid (16.87 mM) had high values; protocatechuic acid (15.71 mM) had a similar value as well. The highest values (above 20 mM) were found for methoxyphenols: eugenol (23.60 mM) and guaiacol (25.87 mM), excluding the aforementioned vanillin; the very highest was determined for gentisic acid (27.44 mM). Thus, benzoic acids had the lowest and highest AC values (Table 1) – from 0.19 to 27.44 mM. From these results and also an earlier study (Švestková et al., 2019), it is clear that the AC is influenced by the resulting overall structure of the substance itself, rather than just part of the structure, i.e. which derivative it is.

### 3.2 Antioxidant capacity of a mixture of two substances

For the mixture of two substances, it was always a 1:1 ratio, with 2 g/l being a total concentration as well, where each substance therefore had 1 g/l. The lowest AC was found for the combination of malic acid and 4-hydroxybenzoic acid (0.72 mM), i.e., the substances individually showing the lowest AC. Even though these were the weakest antioxidants, their resulting AC was much higher than the average (0.25 mM), a significant synergistic effect that amounted to a 189.2% (i.e. 2.9 times) increase in final AC (Table 1).

On the contrary, the highest AC was measured for the mixture of gentisic acid and eugenol (29.07 mM), and the combination of the two most potent antioxidants, gentisic acid and guaiacol (28.47 mM), showed practically the same value. While a synergistic effect was observed in both cases (an increase in AC of 13.9% and 6.8%, i.e. 1.13 and 1.07-fold, respectively), it was very small.

Eugenol, as the substance achieving the highest AC in combination with another, can also be used for its considerable synergistic effect with antibiotics, where it inhibits microorganisms up to 1000 times more than antibiotics alone (Ulanowska & Olas, 2021), thus it is a substance with not only high AC effects but also antimicrobial ones.

**Fig. 3:**
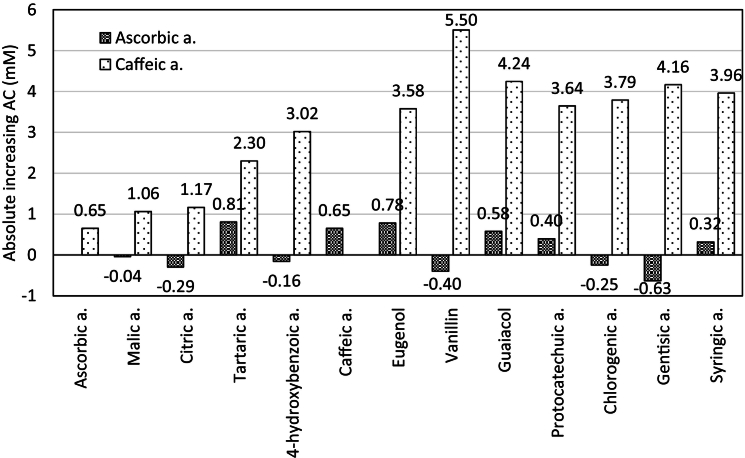
Increase/decrease in AC in the real mixture compared to the theoretical mixture with only additive effect for caffeic acid and ascorbic acid, at concentration 2 g/l.

The absolute decreases/increases in AC as such for the 2 g/l solutions ranged from −0.63 mM (gentisic acid with ascorbic acid) to +5.50 mM (vanillin with caffeic acid) compared to the theoretical mixture with only additive effect (Fig. 3). Caffeic acid with other substances showed an average increase of 3.09 mM; analogously it was 2.19 mM for eugenol, 2.11 mM for guaiacol, 1.93 mM for syringic acid, 1.86 mM for gentisic acid, 1.82 mM for vanillin, 1.84 mM for 4-hydroxybenzoic acid, 1.81 mM for protocatechuic acid, 1.72 mM for chlorogenic acid, 1.29 mM for tartaric acid, 0.85 mM for citric acid, 0.53 mM for malic acid and only 0.15 mM for ascorbic acid.

**Fig. 4:**
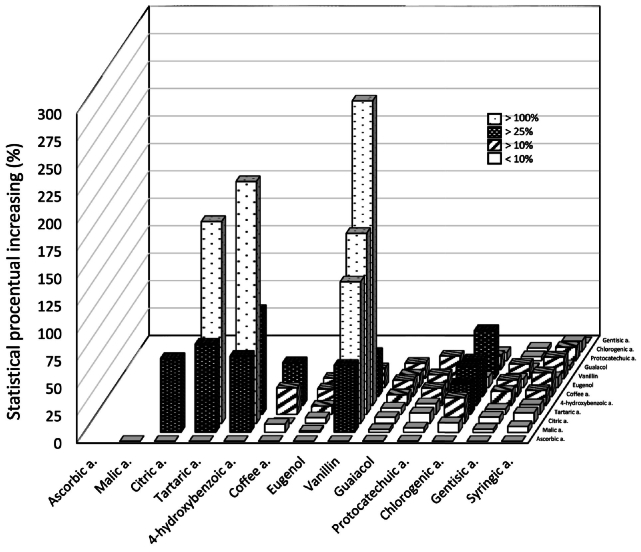
Statistical percentage increase, where each group represents: (A) a statistical percentage increase above 100%, (B) a statistical percentage increase above 25%, (C) a statistical percentage increase above 10%, (D) a statistical percentage increase below 10%, or an antagonistic effect, at concentration 2 g/l.

How many times the AC of the real mixture rises above the theoretical one or in statistical percentage increase (Fig. 4) instead of absolute values, which are also dependent on the initial concentration of the substance. This eliminated the significant difference in AC between the individual pure substances and also the effect of different concentrations.

### 3.3 Antagonistic effect

In addition to the synergistic effect, an antagonistic effect was also observed, i.e. when the resulting AC of the mixture was lower than the average of the 2 substances. Antagonistic effect was observed only for ascorbic acid in combination with: malic acid (−0.7%), 4-hydroxybenzoic acid (−2.4%), chlorogenic acid (−2.5%), gentisic acid (−3.1%), citric acid (−4.5%) and vanillin (−5.9%). The antagonistic effect was observed only in 6 combinations out of 78 and reached max. 6%; it was statistically significant (higher decrease than the magnitude of the standard deviations) only once, observed for gentisic acid.

This antagonistic effect may be caused by the kinetics of vanillin, in a previous study (Soural et al., 2022) it was shown that vanillin exhibits autocatalytic properties and also its antioxidant effect is highly dependent on the reaction time (10 min. vs. 2 min., when in the 2 min. it has up to 73% less absorbance than in 10 min.). Caffeic and syringic acids were also monitored in detail in a previous study, when syringic acid reacted very quickly (without signs of autocatalysis) and caffeic acid did show autocatalytic effects, but its antioxidant effect was not as dependent on the reaction time as vanillin (absorbance in 2 min. is only 3% smaller than in 10 min.). If ascorbic acid preferentially reacted with the FRAP agent in the two-component mixture and at the same time the products did not catalyse the reaction of vanillin with the FRAP agent, this would mean a decrease in the antioxidant properties of vanillin and thus an antagonistic effect.

In other studies, caffeic acid showed antagonistic effects with catechin and quercetin. Similar to vitamin C (ascorbic a.) with other substances, vitamin A (*α*-tocopherol) showed an antagonistic effect as well with caffeic acid and with rosmarinic acid (Peyrat-Maillard et al., 2003; Sonam & Guleria, 2017), provitamin A (*β*-carotene) showed both synergistic and antagonistic effects (Swada et al., 2016). Also, Katalinic (2015) observed an antagonistic effect (by 10.0%) for the binary combination (resveratrol and caffeic acid). For the three-component mixtures, there were decreases in AC of about 20% for rutin, caffeic acid and rosmarinic acid; for rutin, rosmarinic acid and gallic acid; and for rutin, chlorogenic acid and caffeic acid (Hajimehdipoor et al., 2014).

### 3.4 Synergistic effect – multiple increase

The largest fold increase in AC, i.e., the ratio synergistic effect, was observed for tartaric acid with 4-hydroxybenzoic acid, where the resulting AC of the real mixture was 8.9 times higher than the AC of the theoretical mixture without synergistic effect (but this value was burdened by a larger measurement error – see statistical percentage growth), for 4-hydroxybenzoic acid with vanillin and citric acid reached 5.3 and 5.0-fold, respectively, followed by tartaric acid with citric acid and vanillin reaching 3.6-fold, vanillin with citric acid 3.2-fold, and 2.9 to 2.0-fold was observed for malic acid with 4-hydroxybenzoic acid, tartaric acid, citric acid and vanillin. Thus, the increase above 3-fold was in 6 combinations, between 2 and 3-fold in 4 combinations, between 1.25 and 2-fold in 11 combinations, between 1.10 and 1.25-fold in 35 combinations and up to 1.10-fold in 16 combinations.

A synergistic effect was observed in 72 combinations out of total 78; it was statistically significant (higher decrease than the magnitude of the standard deviations) in 67 combinations.

In other studies, caffeic acid showed synergistic effects in a binary mixture with rosmarinic acid (Peyrat-Maillard et al., 2003; Sonam & Guleria, 2017). Vitamin E had synergistic effects (Shi et al., 2007). In another study, more than two-fold AC values were achieved by the binary mixture (gallic acid with caffeic acid) with 137.8% increase, while for the multi-component mixtures there were increases of about 60% (quercetin, gallic acid and caffeic acid / quercetin, gallic acid and rutin) (Hajimehdipoor et al., 2014).

### 3.5 Synergistic effect – statistical percentage increase

In addition to the statistically confirmed increase (Table 1) the statistical percentage increase was calculated (Fig. 4), i.e. by how much the AC increased conclusively in percentage (above the measurement error), not that there is just an increase in AC.

A statistical percentage increase above 100% was observed for the mutual combinations of four substances: citric acid, tartaric acid, 4-hydroxybenzoic acid and vanillin, where five out of six possible combinations showed a statistical percentage increase above 100%. Vanillin with citric acid (by 129%), with tartaric acid (by 165%) and with 4-hydroxybenzoic acid (by 277%), which was the largest statistical percentage increase; citric acid with tartaric acid (by 184%) and with 4-hydroxybenzoic acid (by 220%) (Fig. 2). The only combination below 100% was identified for tartaric acid with 4-hydroxybenzoic (by 95%), almost by 100% as well (Fig. 4).

Further, a statistical percentage increase in AC of >25% was found for malic acid in a mixture with all of these four substances (by 61% to 81%); chlorogenic acid with three of these substances in addition to citric acid (only by 23%); and for caffeic acid with two of these substances; except for citric acid where there was no statistical increase (n. s.) and tartaric acid (only by 24%). Apart from the above, it was only observed for the combination of chlorogenic acid with caffeic acid (by 33%).

A statistical percentage increase in AC of 10% or more was observed for syringic acid in six of the 12 combinations (6/12), protocatechuic acid (6/12), guaiacol (5/12), eugenol (5/12), gentisic acid (3/12), and only ascorbic acid was not detected (0/12). Apart from the instances of statistical increases above 25% or 100%, an increase was also observed for the already mentioned substances: citric acid (2/12), tartaric acid (2/12), 4-hydroxybenzoic acid (5/12), caffeic acid (6/12), vanillin (5/12) and chlorogenic acid (4/12), only for malic acid was it not observed (0/12).

According to research by Skroza et al. (2022) measuring equimolar mixtures of hydroxybenzoic acid measured using FRAP, synergistic effect was confirmed in a large number of various mixtures. Mixtures containing gentisic acid had a synergistic effect (28%–89% difference). The mixture of protocatechuic acid and syringic acid showed only additive effect.

Thus, vanillin and 4-hydroxybenzoic acid showed a statistical percentage increase of 10% or more with all but one substance (11/12), similarly caffeic acid (9/12), chlorogenic acid (8/12), tartaric acid and citric acid (7/12), as well as substances with an observed statistical increase of up to 25%: syringic acid and protocatechuic acid (6/12), guaiacol and eugenol (5/12), few combinations with statistical synergistic effect of 10% or more were shown by malic acid (4/12), gentisic acid (3/12), and no combination for ascorbic acid (0/12).

### 3.6 Synergist or antioxidant

**Fig. 5:**
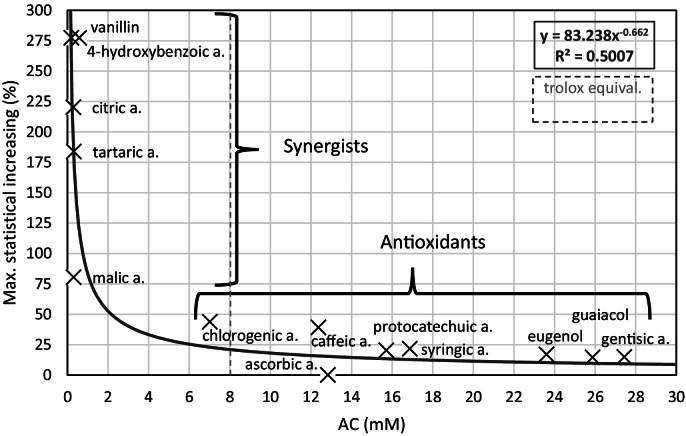
Dependence of the maximum statistical increase in antioxidant capacity (%) in the mixture on the antioxidant capacity of the pure substance (mM), at concentration 2 g/l.

Based on the AC of the individual substances and the relative synergistic effects, it is better to classify a particular substance as a synergist or antioxidant (although this is not an absolute classification). In general, substances with low AC showed higher synergistic effects and vice versa (Fig. 5, Table 1). The best synergistic effects were exhibited by: vanillin, 4-hydroxybenzoic acid, tartaric acid, citric acid (with max. 277% statistical percentage increase), where vanillin and 4-hydroxybenzoic acid had a demonstrable synergistic effect with almost all tested substances. These substances individually had low AC (much below 8 mM, corresponding to 2 g/l Trolox), thus they can be classified as synergists together with malic acid (Fig. 5).

On the other hand, substances with very high antioxidant capacity above 15 mM at the same concentration of 2 g/l, i.e. gentisic acid, guaiacol and eugenol, protocatechuic acid and syringic acid showed the lowest increases within the synergistic effect (max. 22% statistical percent increase - in order max. 15%, max. 15%, max. 17%, max. 20% and max. 22%). Also, substances with increased antioxidant capacity such as chlorogenic acid and caffeic acid (7.0 mM and 12.3 mM) had low but slightly higher synergistic effects (max. 44% and 39% statistical percentage increase); these substances can be classified as antioxidants together with ascorbic acid (Fig. 5).

Shifting between groups can be observed, e.g. for vanillin, ascorbic acid and 4-hydroxybenzoic acid. Vanillin belonging to the methoxyphenol derivatives is structurally similar to eugenol (23.60 mM, max. 17%) and guaiacol (25.87 mM, max. 15%), which are antioxidants (Fig. 5), but its properties are synergistic (0.58 mM, with max. 277%); a possible reason for this phenomenon is the presence and position of the aldehyde group in vanillin. On the other hand, ascorbic acid belonging to the carboxylic acids is structurally similar to malic acid (0.31 mM, with max. 81%), citric acid (0.29 mM, with max. 220%) and tartaric acid (0.32 mM, with max. 184%), but its AC properties make it an antioxidant (12.83 mM, less than max. 1%), with the possible reason for this shift being the presence of a cyclic structure in ascorbic acid. Another substance showing a shift is 4-hydroxybenzoic acid, which structurally belongs to phenolic acids together with gentisic acid (27.44 mM, max. 15%), protocatechuic acid (15.71 mM, max. 20%), syringic acid (16.87 mM, max. 22%), caffeic acid (12.36 mM, max. 39%) and chlorogenic acid (7.01 mM, max. 44%), but instead of antioxidant properties it has synergistic properties (0.19 mM, max. 277%), where a possible reason for this shift is only one hydroxyl group (phenol) instead of two as in the case of other acids (polyphenols), or the absence of a methoxy group in the case of syringic acid. Furthermore, the slightly lower AC of chlorogenic acid may be due to the fact that it is a glycoside of caffeic acid, where for the same 2 g/l concentration, taking into account molar masses, the AC would correspond to 13.8 mM (for pure caffeic acid without the carbohydrate part in chlorogenic acid), i.e. a value approximately that of caffeic acid itself (12.3 mM).

The maximum statistical percentage increase (%) was plotted as a function of the antioxidant capacity of the pure substance (mM), where such a dependence is relatively well interleaved by a power regression curve (R^2^ = 0.5007), despite the fact that each measurement is to some extent burdened by a different measurement error. In this chart it is clearly visible which substances show rather antioxidant or synergistic properties (Fig. 5). The monitored substances increasing AC by >75% and with antioxidant capacity up to 0.6 mM are rather synergists and substances increasing AC up to 50% with antioxidant capacity above 5 mM are rather antioxidants. Note: AC of Trolox, as a synthetic antioxidant of the same 2 g/l concentration, corresponds to 8 mM. The hypothesis that a synergist can extremely increase AC was confirmed by an increase of up to 890% in a binary mixture. The highest absolute increase in AC was +5.5 mM for the binary mixture with only 2 g/L, the increase alone thus corresponding to 1.375 g/L of Trolox.

### 3.7 Eliminating the “FRAP background” effect

To rule out the “FRAP Background” effect, in which the FRAP measuring solution itself slowly increases absorbance after preparation, by 0.0109 in 6 h (Soural et al., 2022), a check was performed to see if this effect was present in the AC increase. This would increase the resulting AC by only 0.022 mM, which is virtually no increase relative to values of real mixtures from 0.72 mM (Fig. 1) to 29.07 mM, which were measured sequentially after measurement of the pure substances (i.e. within 6 h).

### 3.8 Effect of double diluted sample on final AC value

For pure substances, AC was determined in parallel from measurements of doubly-diluted (DD) samples. The AC values from this additional measurement were compared with the initial AC values, where the substances were of twice the concentration - as single-diluted (SD). Due to the measurement error of each substance (standard deviation) and the repetition of the calibration curve, as is known for FRAP in the previous study (Soural et al., 2022), the ratio of the determined antioxidant capacities SD: DD is not equal to 1, as an optimal state.

For most substances, the ratio SD: DD was in the interval 0.94–1.08, i.e. AC values from single dilution are smaller by up to 6% or larger by up to 8% than the values  obtained by double dilution. This is comparable to the standard deviations from SD (Table 1, Fig. 1b), which in relative size are up to 2%.

**Fig. 6:**
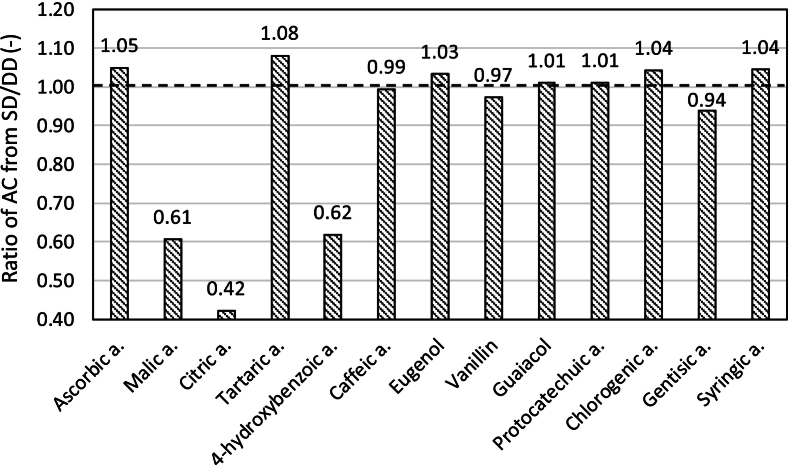
The ratio of the determined AC by single-diluted (SD) to doubly-diluted (DD) samples of pure substances (SD: DD).

Only in the case of 4-hydroxybenzoic acid, malic acid and citric acid, with the lowest AC, was the ratio SD: DD considerably lower than 1, i.e. 0.62; 0.61 and 0.42. The reason is apparently the standard deviations, which were >25% (25.6–65.8%) for these substances (Table 1, Fig. 1a, Fig. 2), within the SD determination (Fig. 6).

### 3.9 Synergistic effect in food

Some of the phenolic substances (gentisic acid, caffeic acid, syringic acid, chlorogenic acid, eugenol and guaiacol) that were subject to measurement can be used for fortification of apple/orange juice, where they increase the antioxidant capacity of the juice. The largest percentage increase in AC was obtained for gentisic acid in apple juice, with a 43.5% increase in the antioxidant capacity of the juice, followed by guaiacol (35.0%), eugenol (32.9%), syringic acid (25.5%), caffeic acid (20.2%) and chlorogenic acid (11.4%) for the same doses of 40 mg/l and the same measurement method, i.e. FRAP (Soural et al., 2020).

For example, the application of various combinations of foods classified as fruit, vegetables and legumes showed a synergistic effect in 21% of the cases, an antagonistic effect in 25% and only an additive effect in 54% of the cases (Wang et al., 2011).

Apple juice, which is not as flavourful as orange juice, becomes very attractive when fortified with eugenol or vanillin (e.g. at 40 mg/l). Both of these substances are key components of the aroma (Ferreira & Lopez, 2019; Motooka et al., 2015) and thus give apple juice its aroma but also its taste of vanilla or clove. Initial sensory evaluations indicate highly increased attractiveness of these juices, referred to by consumers, with some exaggeration, as “Christmas drinks”. Some foods fortified purely for sensory purposes may also have an increased nutritional benefit, e.g. an unexpected increase in AC, especially through a synergistic effect.

Citric acid exhibited a synergistic effect with oxalic acid in the context of antibrowning effect in foods (Son et al., 2001).

Not only the addition of pure substances but also the combination of the two foods can cause a synergistic effect, e.g., smoothies made of sour cherry and quince, where the AC of both juices was around 28 mM, reaching 33.2 mM, i.e., 16.3% more, within the FRAP method in a 1:1 mixture (Nowicka et al., 2017).

## 4 Conclusion

Antioxidant capacity (AC) is the result of the interaction of all substances in a given biological matrix. The pure substances alone may not exhibit significant AC, but can be significant synergists that will greatly increase the AC of the mixture when combined with other substances. For 13 monitored substances occurring in foods, members of carboxylic acids, phenolic acids and methoxyphenols, AC was measured by FRAP in Trolox equivalents, alone and in all 78 combinations within binary mixtures. The resulting AC of the real mixtures increased by up to +5.5 mM for the synergistic effect and decreased by −0.6 mM for the antagonistic effect at a concentration of 1 g/l for both substances in the mixture. The individual AC changes were converted to multiples of the AC increase so that the results were not dependent on the original concentration, with the maximum increase up to 8.9 times and the decrease down to 0.94 times. To exclude measurement error, the statistical percentage increase was further determined, i.e. by how much AC increased when random errors were eliminated, where an increase in AC of up to 277% was calculated. Substances with high AC were found to have a small synergistic effect (statistical percentage increase) and vice versa. Among the substances studied, carboxylic acids – malic acid, tartaric acid and citric acid – as well as 4-hydroxybenzoic acid and vanillin can be included among the synergists; on the other hand, methoxyphenols eugenol and guaiacol as well as phenolic acids – gentisic acid, protocatechuic acid, syringic acid, chlorogenic acid and caffeic acid – as well as ascorbic acid can be included among antioxidants with small synergistic effect. The determination of the final AC from variously diluted samples can be burdened by measurement error, where for substances with very low AC values it can be tens of percent, but for antioxidants that are the subject of measurement it is only up to 8%. To increase AC by means of not just synergistic effect, foods can be fortified with sensory pleasant vanillin or eugenol providing a vanilla/clove aroma or also vitamin C, i.e. ascorbic acid.

## Ethical approval

This article does not contain any studies with human participants or animals performed by any of the authors.

## CRediT authorship contribution statement

**Petra Švestková:** Resources, Methodology, Investigation, Conceptualization. **Josef Balík:** Writing – review & editing, Supervision. **Ivo Soural:** Writing – original draft, Data curation.

## Declaration of competing interest

The authors declare that they have no known competing financial interests or personal relationships that could have appeared to influence the work reported in this paper.

## Data Availability

Data will be made available on request.
